# The Sesquiterpene Biosynthesis and Vessel-Occlusion Formation in Stems of *Aquilaria sinensis* (Lour.) Gilg Trees Induced by Wounding Treatments without Variation of Microbial Communities

**DOI:** 10.3390/ijms151223589

**Published:** 2014-12-18

**Authors:** Zheng Zhang, Jianhe Wei, Xiaomin Han, Liang Liang, Yun Yang, Hui Meng, Yanhong Xu, Zhihui Gao

**Affiliations:** 1 National Engineering Laboratory for Breeding of Endangered Medicinal Materials, Institute of Medicinal Plant Development, Chinese Academy of Medical Sciences and Peking Union Medical College, Beijing 100193, China; E-Mails: zhangzheng@implad.ac.cn (Z.Z.); xmhan@implad.ac.cn (X.H.); liangliang@implad.ac.cn (L.L.); yhxu@implad.ac.cn (Y.X.); zhgao@implad.ac.cn (Z.G.); 2 Hainan Provincial Key Laboratory of Resources Conservation and Development of Southern Medicine, Hainan Branch of the Institute of Medicinal Plant Development, Chinese Academy of Medicinal Sciences and Peking Union Medical College, Wanning 571533, China; E-Mails: yangyun43@aliyun.com (Y.Y.); huiziqq@163.com (H.M.)

**Keywords:** *Aquilaria sinensis*, wound, microbe, vessel occlusion, sesquiterpene, plant defense response

## Abstract

As widely recognized, agarwood formation in *Aquilaria* trees is induced by external wounding. Because agarwood usually harbors specific microbes, the function of microbes in agarwood formation has been debated for almost a century. In this study, two wounding methods, the burning-chisel-drilling method (BCD) and the whole-tree agarwood-inducing method (Agar-Wit), were used under the non-contamination of environmental microorganisms. After pyrosequencing the small rRNA subunits of the wounds induced by the BCD and Agar-Wit, no substantial variation was observed either in fungal and bacterial enrichment and diversity or in the relative abundances of taxa. By contrast, significant variations in fungal and bacterial communities were detected following the partial tree pruning (PTP)-wounding. The wound-induced sesquiterpene biosynthesis and vessel-occlusion formation, however, were found to be similar in all types of wounded trunks. We thus infer that wounding in the absence of variations in microbial communities may induce agarwood formation. This result does not support the long-standing notion that agarwood formation depends on microbes.

## 1. Introduction

Plants, as sessile organisms, face many stress situations, and their growth and productivity are adversely affected by nature’s wrath in the form of various abiotic and biotic stress factors [1]. To cope with these stresses and be adaptable to environments, plants use their secondary metabolites, and thus present an important source of active pharmaceuticals, but at a very low or even undetectable yield. Because these metabolites can protect plants from attacks by insects, herbivores and pathogens, or make plants survive other biotic and abiotic stresses, various culture strategies have been developed to improve the yield of such metabolites [2].

Agarwood is widely used in traditional medicines, such as a digestive, sedative, and antiemetic drug, and it is also popular as an incense and perfume in the Middle East, South Asia, Japan, and China. The main chemical compounds present in agarwood are sesquiterpenoids and phenylethyl chromone derivatives [3].

These compounds are not detectable in the fresh parts of healthy *Aquilaria* wood, but might form in wounded or microbe-infected *Aquilaria* trees. The accumulation of phytoalexins (sesquiterpenes) and formation of physical barriers (vesselocclusions) were speculated to contribute to the chemical inhibition and physical restriction of microbes within vessels to prevent their spread [4]. Because agarwood usually contains specific microbes, it is considered to be a pathological product generated as a result of fungal invasion of the host [5,6]. Agarwood formation has been studied since 1929, and the agar zones have been reported to be associated with mold- and decay-related fungi [7,8]. The common fungi that infect *Aquilaria* spp. have been identified, including *Aspergillus* sp. [9], *Epicoccum granulatum* [10], *Cytosphaera mangiferae* [11], *Fusarium oxysporum* and *Chaetomium globosum* [12], and *Fusarium* sp., *Trichoderma* sp., and *Lasiodiplodia* sp. [13]. However, agarwood formation was also reported not to depend on the activity of specific fungi, but represent a general reaction of the host to injury or invasion [14,15,16]. A recent study showed that chemical wounding (using solutions of FeCl_2_, NaCl, or NaHSO_3_), instead of fungal inoculations, induced agarwood formation effectively [17].

Our previous research showed that the Agar-Wit, a whole-tree agarwood-inducing technique, could induce agarwood formation in the branches several meters distant from an infused trunk [18,19]. So far, no evidence has been found to demonstrate whether wounding or fungal invasion plays a dominant role in agarwood formation.

The Ion Torrent Personal Genome Machine (PGM; Life Technologies, Guilford, CT, USA) platform, because of its low cost per sequencing run, has been widely used for sequencing bacteria [20,21,22], as well as for sequencing Internal Transcribed Spacer Region 1 (ITS1) of fungi [23,24]. Although numerous fungi have been isolated from agarwood, no complete picture of the microbial community structure during agarwood formation is now available, because microbiome studies only focus on one or several microbial domains at one time. To determine the distinction of microbial domains in the agarwood induced by different wounding treatments, we used the Ion Torrent PGM platform and obtained the microbial taxonomic profiles by comparing the outcomes of the two treatments. Meanwhile, we analyzed the sesquiterpene components and the vascular occlusions of the agarwood induced by the three wounding treatments.

## 2. Results

### 2.1. Fungal Diversity Did not Vary in the Stem of A. sinensis Treated by BCD and Agar-Wit Methods

**Figure 1:**
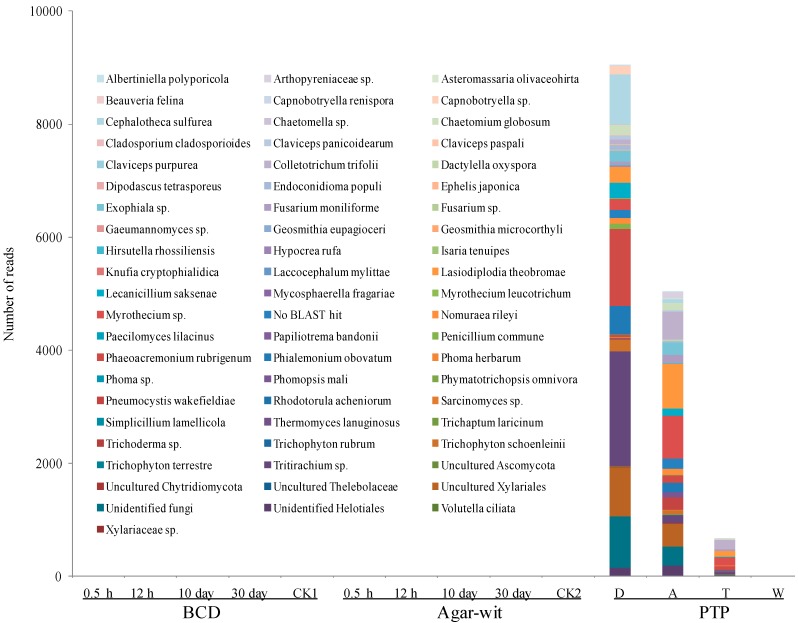
Distribution of fungal OUTs at the species level in the wounded wood induced by three different wounding methods, BCD, Agar-Wit and PTP. The wounded wood obtained by the partial-trunk-pruning (PTP) method was divided into four zones,* i.e.*, the decay (D), agarwood (A), transition (T), and white (W) zones. Samples obtained from the untreated healthy trees and the trees treated by the Agar-wit with ddH_2_O not inducer are referred to as CK1 and CK2, respectively.

To prevent the invasion of environmental microbes, *Aquilaria* trees were induced to produce agarwood in a closed system by a typical physical wounding method, the burning-chisel-drilling (BCD) treatment, in which a burning and red-hot iron nail (approximately 600 °C) was inserted into the *Aquilaria* tree trunk. This high-temperature treatment not only induced agarwood formation around the hole, but also killed microbes and thus sterilized the hole surface. Moreover, to prevent the invasion of microorganisms, this hole was immediately sealed with sterilized paraffin wax. Next-generation sequencing (Ion Torrent) was adopted to measure fungal biodiversity of the agarwood samples collected 0.5 h, 12 h, 10 days and 30 days after the BCD treatment. At the early, middle, and late stages after the BCD treatment, no fungi were detected in the samples; similarly, no fungi were detected in the control CK1 samples (Figure 1). To avoid any contamination from the environmental microorganisms, the Agar-wit treatment was applied with a sterilized agarwood inducer, and the infusion device was handled using the aseptic techniques. Once again, at the early (0.5 and 12 h), middle (10 days), and late stages after the Agar-wit treatment, no fungi were detected. Similarly, no fungus was detected in the control CK2 samples (Figure 1). By contrast, numerous fungi were detected in the agarwood induced by the partial-trunk-pruning (PTP) method. The number of reads recovered from the decay, agarwood, transition, and white zones decreased sharply, being 9095, 5044, 663 and 0, respectively. Sixty-three species obtained from the decay zone were mostly nonpathogenic environmental microorganisms. In the agarwood and transition zones, however, plant pathogens, such as *Lasiodiplodia theobromae*, *Myrothecium* sp., and* Colletotrichum trifolii* were over-presented, constituting nearly 50% of the fungal communities of these two zones (Figure 1).

### 2.2. Bacterial Diversity Did not Vary in the Stem of A. sinensis Treated by BCD and Agar-Wit Methods

**Figure 2:**
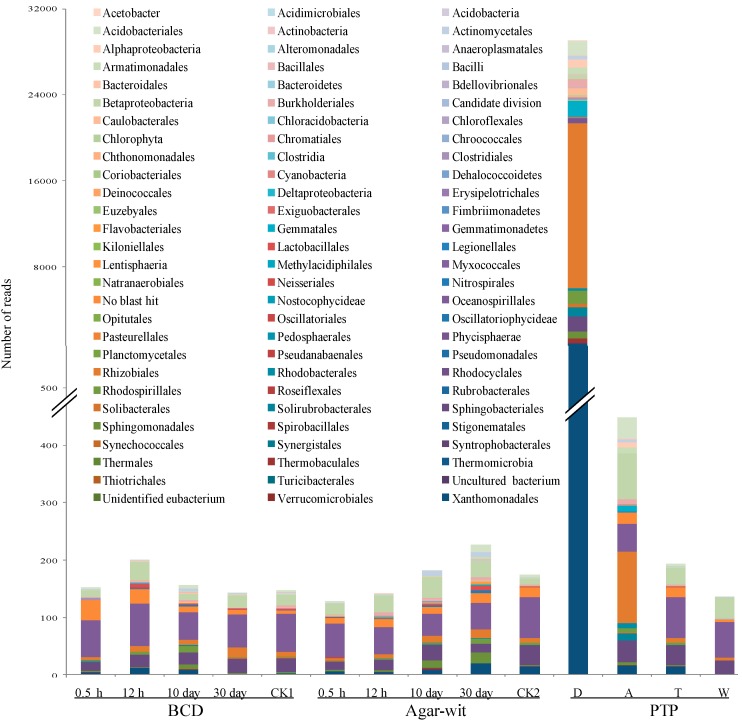
Distribution of bacterial OUTs at the ordinal level in the agarwood induced by three different wounding methods, BCD, Agar-Wit and PTP. The wounded wood obtained by the partial-trunk-pruning (PTP) method was divided into four zones, the decay (D), agarwood (A), transition (T), and white (W) zones. The samples obtained from the untreated healthy trees and the trees treated by the Agar-wit with ddH_2_O not inducer are referred to as CK1 and CK2, respectively.

Bacterial biodiversity of the agarwood samples was also measured 0.5 h, 12 h, 10 days and 30 days after the BCD treatment. At the early stages (0.5 and 12 h), 152 and 201 reads were respectively recovered; at the middle stage (10 days), 157 reads were recovered; at the late stage (30 days), 143 reads were recovered. Fifty-nine species-level and 33 ordinal-level operational taxonomic units (OTUs) were observed; 72.6% of the species-level OTUs were members of the beta- and gamma-proteobacteria subclasses, which have also been reported to be dominant bacteria in other plants [25,26]. In CK1 samples, 147 reads were identified to belong to the beta- and gamma-proteobacteria subclasses (73.8%). No statistically significant difference was observed in bacterial biodiversity at different time points in the BCD treatment and the control samples (Figure 2). Moreover, no statistically significant difference was observed in bacterial diversity at different time points in the Agar-wit treatment and the control samples (Figure 2). Thirty minutes, twelve hours, ten days, and thirty days after the Agar-wit treatment recovered 128, 142, 183 and 227 reads, respectively. Seventy-one species-level and 39 ordinal-level OTUs were detected in the Agar-wit-treated tree trunks, and the beta- and gamma-proteobacteria (>60%) were again found to be the most dominant subclasses. In the CK2 samples, 175 reads were determined to belong to the beta- and gamma-proteobacteria subclasses (57.8%). Comparison of these results showed that the agarwood induced by the PTP had more bacteria, with 29,067, 448, 194, and 136 reads respectively found in the decay, agarwood, transition, and white zone (Figure 2). In the PTP-treated tree trunks were detected 310 species-level and 83 ordinal-level level OTUs, and >70% of the species-level OTUs belonged to the orders of *Rhizobiales*, *Gemmatales*, *Sphingobacteriales*, *Acidobacteriales*, and *Rhodospirillales*, the most abundant bacteria in soil [27].

### 2.3. Three Different Types of Wounding All Induced Vessel-Occlusion Formation and Sesquiterpene Biosynthesis

Because significant differences had been observed in bacterial and fungal diversity in the wounded wood samples obtained from the three different wounding treatments, we sought to test whether the samples induced by the BCD and Agar-Wit treatments produced similar components and structures as the samples induced by the PTP. We therefore examined the chemical components and anatomy of these wounded wood samples.

**Figure 3:**
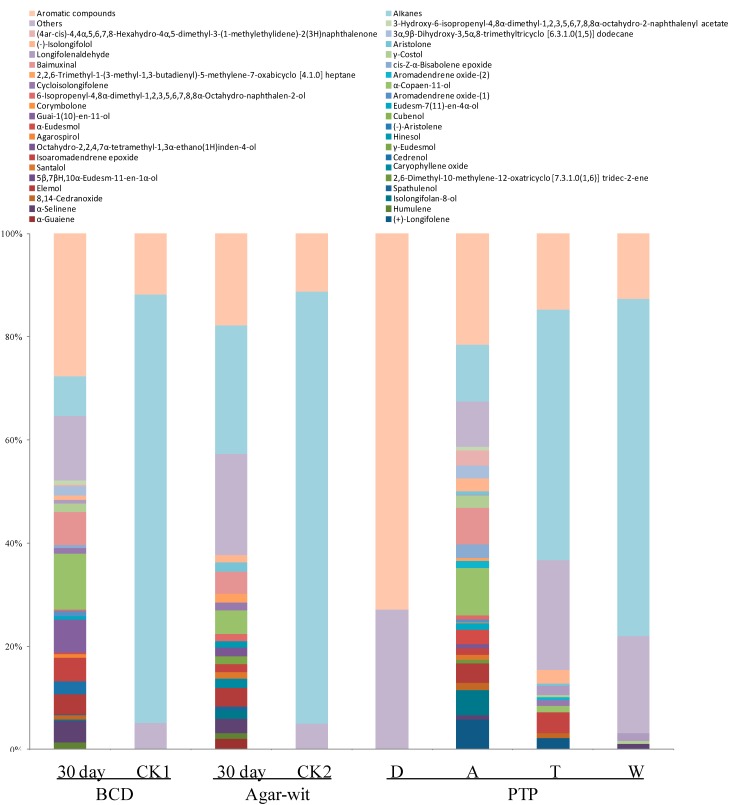
Type and content of essential oils in the agarwood samples obtained by the three different wounding methods, BCD, Agar-Wit and PTP. The wounded wood obtained by the partial-trunk-pruning (PTP) method was divided into four zones, the decay (D), agarwood (A), transition (T), and white (W) zones. The samples obtained from the untreated healthy trees and the trees treated by the Agar-wit with ddH_2_O not inducers are referred to as CK1 and CK2, respectively. Relative amount (%) was defined to be the peak area relative to the total peak area.

Our results showed that the wounded wood induced by the BCD and Agar-wit and by the PTP (Zone A) had essential oils with similar components. Forty-seven components were identified in the agarwood induced by the BCD, making up 80.40% of the total volatiles, the major constituents of which were such sesquiterpene compounds (37.68%) as α-copaen-11-ol (4.63%), baimuxinal (4.31%), elemol (3.64%), α-selinene (2.81%), and α-guaiene (2.12%) (Figure 3). Forty-four components were identified in the agarwood induced by the Agar-wit, making up 87.51% of the total volatiles. The predominant compounds in the essential oil of the Agar-wit-induced agarwood were sesquiterpenes (52.12%), including α-copaen-11-ol (10.84%), baimuxinal (6.36%), guai-1(10)-en-11-ol (6.35%), isoaromadendrene epoxide (4.58%), α-selinene (4.08%), elemol (3.95%), and cedrenol (2.43%) (Figure 3). CK1 and CK2 samples had no such essential oils like in the Agar-wit and BCD treatments, but they were rich in alkanes, respectively reaching 79.3% and 83.8% those of the Agar-wit and BCD treatments (Figure 3). In the agarwood induced by the PTP, the number and content of sesquiterpene compounds showed a normal distribution in terms of distance from the wound surface. In the decay zone harboring the richest microbial community existed no sesquiterpenes, while the agarwood zone featured the highest relative abundance of sesquiterpenes and contained a total of 26 sesquiterpene compounds (59.26%), with α-copaen-11-ol (9.14%) and baimuxinal (7.01%) being the predominant sesquiterpenes in the essential oil. In the transition and white zones, the type and relative abundance of sesquiterpenes sharply decreased along with the reduction in species number and microbe amount, where 10 and 3 sesquiterpenes were identified, respectively, making up 15.39% and 3.17% of the essential oils.

**Figure 4:**
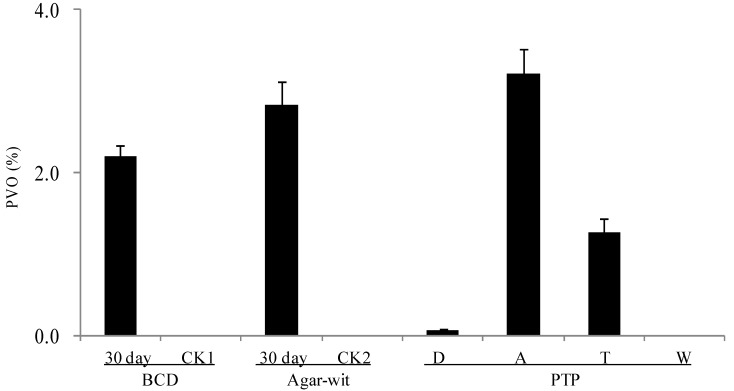
Wound-induced vessel occlusions detected (in the decay, agarwood, transition, and healthy zones) at least 10 months after the PTP treatment and 30 days after the Agar-wit and BCD treatments. No tyloses were detected in any vessel lumen of an unwounded control sample. PVO means the percentage of vessels with occlusions per mm^2^. Error bars represent the SE, *n* = 5. The wounded wood obtained by the PTP method was divided into four zones, the decay (D), agarwood (A), transition (T), and white (W) zones. The samples obtained from the untreated healthy trees and the trees treated by the Agar-wit with ddH_2_O not inducers are referred to as CK1 and CK2, respectively.

The formation of vessel occlusions was postulated to be closely correlated with the sesquiterpene components of the agarwood formed [4]. On Day 30, the percentage of vessels with occlusions (PVO) reached 2.21 in the agarwood induced by the BCD and 2.4 in the agarwood induced by the Agar-wit, but was undetectable in the CK1 and CK2 samples (Figure 4). The PVO in the agarwood induced by the PTP showed a normal distribution in terms of distance from the wound surface, respectively being 0.27, 3.21, 1.27 and 0 in the four zones. The PVO decreased along with the reduction in species number and microbe amount, as well as in sesquiterpene content.

## 3. Discussion

In this study, the Ion-Torrent sequencing method was used to test the communities of bacteria and fungi in the wood treated by two wounding methods BCD and Agar-wit, under the non-contamination of external microbes. From 0.5 h to 30 days after the treatments, there was no significant variation of bacterial and fungal community structures. On day 30 were detected sesquiterpene biosynthesis and vessel-occlusion formation. The results indicate that the wounding without invasion of external microbes can induce the sesquiterpene biosynthesis and vessel-occlusion formation. To our knowledge, this is the first report that wounding in the absence of variations in microbial communities can induce agarwood formation in *A. sinensis*.

To confirm the hypothesis that agarwood is induced by wounding but not by microbes [28], preventing the invasion of external microbes is a prerequisite in the process of wounding treatment. Thus, we used both the typical physical wound (BCD) and chemical wound (Agar-wit) methods to induce *Aquilaria* trees to produce agarwood. The BCD treatment at a very high temperature not only induced the sesquiterpene biosynthesis and vessel-occlusion formation around the hole, but also killed microbes, consequently sterilizing the hole surface. In the Agar-wit treatment, a sterilized agarwood inducer was used and the infusion device was handled using aseptic techniques.

Because endophytic microbes cannot be moved from live wood, the community of endophytic microbe may be changed by wounding, which ultimately causes agarwood formation. To avoid this possibility, we compared the microbial communities of all groups at different time points after wounding. We found no fungi in the wood treated by the BCD and Agar-wit, and observed similar bacteria communities in the wounded and control groups. This excluded the possible effect of internal microbes on agarwood formation.

Is the Ion-Torrent sequencing method reliable? In recent years, this method has been widely applied in determining the microbe biodiversity of soil [23,27], guts [20] and plants [25,26]. In this study, the Ion-Torrent sequencing was used for the first time on *Aquilaria* trees. The wounded samples by the PTP were taken as positive control, in which 14,802 reads at 63 species levels and 29,397 reads at 310 species levels were observed respectively for fungal and bacterial diversity. The results indicate that the pyrosequencing method can be used to detect the presence of fungi and bacteria. We therefore come to the conclusion that the sesquiterpene biosynthesis and vessel-occlusion formation was induced by the BCD and Agar-wit treatment, but not by external and internal fungi and bacteria. Agarwood formation is a slow and continuous process, so it takes a long time for white, soft, wood to turn into dark, hard, and scented resins, agarwood. After wounding, the number of vessel occlusions and the amount of sesquiterpenes keep increasing, till agarwood is ultimately formed in the wounded wood of *A. sinensis*. Thus the accumulation of vessel occlusions and sesquiterpenes is used as the markers for agarwood formation [4]. Here the sesquiterpene biosynthesis and vessel-occlusion formation was tested on day 30 after wounding. The wood treated by the BCD and Agar-wit had both vessel occlusions and essential oil of agarwood similar to those by the PTP, with α-copaen-11-ol and baimuxinal being the predominant sesquiterpenes in all three essential oils and as well as in wild agarwood [18,19].

Agarwood formantion has long remained a mystery. Facing the assumptions of pathological, wounding/pathological, and nonpathological processes [28], most researchers believe that agarwood is a pathological product [4,5,12]. In a natural environment, agarwood formation is most likely caused by external wounds, such as wind breaking, insect biting, and man-intented wound. Entering an agarwood tree through a wound, environmental microbes can easily decompose, subsequently witnessing agarwood formation under a tropic climate. Since 1929, many fungi have been observed and isolated from agarwood samples induced by mechanical wounding, incluing *Botryodiplodia theobromae* [9], *Epicoccum granulatum* [10], *Cytosphaera mangiferae* [11], *Fusarium oxysporum* and *Chaetomium globosum* [12]. In the past four decades, Chinese researchers have made great efforts to screen effective microbes and develop inoculation methods for inducing agarwood formation. In 1976, a typical fungus, *Menanotus flavolives* from the agarwood of *A. sinensis* was proved to accelerate agarwood formation [5]. Then *M. flavolives* was further proved to induce *A. sinensis* to produce brown agarwood, where oxo-agarospirol was detected. These results evidenced the role of microbes in agarwood formation [5]. Even though some microbes from agarwood, such as *Xylaria* sp. and *Lasiodiplodia* sp., were used to induce agarwood, with a somewhat good effect [29,30], no microbes-induced method has served as a dominant method in agarwood production. Currently, wounding methods, like axe wounds, severe bark removal and nailing, are widely applied to produce low-grade agarwood [19].

Rahman and Basak demonstrated that drilling coupled with or without fungal inoculation could induce agarwood formation, but their experiment was done without avoiding the contamination of environmental microbes [14]. Blanchette and van Beek found that the chemical salt injected into a drilled hole via a tube could induce agarwood formation more efficiently than fungi culture, and thus deduced that chemical agents but not fungal cultures or fungal elicitors might disrupt living cells and better induce agarwood formation [17]. Because these wounds were all open to the environments, the possibility of microbial functions could not be completely eliminated in agarwood formation.

In this work, the BCD (physical wound) and Agar-wit (chemical wound) under the non-contamination of microbes were demonstrated to be capable of inducing the sesquiterpene biosynthesis and vessel-occlusion formation individually. We thus hypothesized that physical and chemical wounds as well as microbes’ invasion may activate plant defense response to induce agarwood formation in *Aquilaria* trees. Accordingly, we deduced that all kinds of proper woundings applied on *Aquilaria* wood may induce agarwood formation, and then we investigated the Agar-wit [19]. Nevertheless, our study did not deny the possibility that microbes can induce agarwood formation and may also play an important role in other wounds. What role do they play? How do they affect agarwood formation? Those need to be further studied.

## 4. Experimental Section

### 4.1. Plant Material

To prevent the invasion of environmental microbes, the burning-chisel-drilling (BCD) and whole-tree agarwood-inducing method (Agar-Wit) were adopted to induce agarwood formation. The plant materials used in the experiment were four-year-old *A. sinensis* saplings, which were grown in a greenhouse at the Hainan Branch of the Institute of Medicinal Plant Development (18°44'N, 110°11'E). These saplings were approximately 2.2 m tall, with the stem diameter of 4.0 ± 0.7 cm, at a height of 10 cm above the ground.

#### 4.1.1. Agarwood Induced by BCD Method

In the BCD treatment, three holes were drilled in each trunk, from approximately 30 cm above the ground to the trunk top, with a burning and red-hot iron nail (5 mm in diameter). The drilled holes, approximately 10 cm apart, were immediately sealed with sterilized paraffin wax to prevent microorganism invasion. One month after the drilling, 45-mm-long samples (with the bark removed) were collected around the holes, and the stem sections 25 mm away from the holes were discarded. The residual samples were 20-mm-long. Of these samples, 2-mm-long stems were collected and immediately fixed in FAA for quantifying vascular-occlusion formation; the remaining 18-mm-long stems were immersed in liquid nitrogen and stored at −80 °C for examination of bacterial and fungal diversity as well as for the analysis using GC-MS; the untreated saplings were used as negative control (CK1).

#### 4.1.2. Agarwood Induced by Agar-Wit Method

After sterilizing the trunk surface with 75% alcohol, a 4-mm diameter hole 20-mm deep was drilled in the stem approximately 300 mm above the ground to the trunk top, into which was injected the aseptic agarwood-inducer solution or ddH_2_O via a sterilized transfusion set and by exploiting the transpiration pull. The deep hole was immediately sealed with sterilized paraffin wax to prevent microbes’ invasion. The average data from five saplings (*n* = 5) in combination were statistically analyzed. Five saplings were treated with the agarwood-inducer solution and five received sterile ddH_2_O (CK2). After one month, 50-mm-long samples (with the bark removed) were collected at a height of 15 cm above the hole, and then 2-mm-long stem samples were immediately fixed in FAA for use in quantifying vascular-occlusion formation. The remaining 48-mm-long stem samples were immersed in liquid nitrogen and stored at −80 °C for use in examination of bacterial and fungal diversity as well as for the analysis using GC-MS.

#### 4.1.3. Agarwood Induced by Partial-Trunk-Pruning (PTP) Method

Agarwood samples were collected from a mature *A. sinensis* tree (approximately 10 years old) in Yanfeng, Hainan, China (19°57'N, 110°33'E), and were used as positive control. This tree had been wounded 10–11 months before by the traditional and external (PTP) wounding methods, as previously reported [19]. A 100-mm-long agarwood sample (with the bark removed) was collected from the apical end of the cut stem, and divided into four zones. The first zone under the wound surface was a black and friable zone named D (decay) (25-mm-long); the second zone was a reddish-brown and hard agarwood zone named A (agarwood) (6-mm long); the third zone was a snuff-colored transition zone named T (transition) (30-mm-long); and the last zone was a white-wood zone named W (white) (39-mm-long). Five-mm-long samples were rapidly collected from the middle of each zone and sectioned. Three sections (approximately 4 mm^2^) of each zone were immediately fixed in formalin-acetic acid-alcohol (FAA) and used for quantifying the formation of vascular occlusions. The remaining sections of each zone were immersed in liquid nitrogen and stored at −80 °C for the examination of bacterial and fungal diversity as well as for gas chromatography-mass spectrometry (GC-MS).

### 4.2. Genomic DNA Isolation

The aforementioned wood samples (1.0 g) were powdered using a mortar and pestle in the presence of liquid nitrogen. The genomic DNA was extracted from the powered samples using a DNeasy Plant Maxi Kit (Qiagen, Valencia, CA, USA) according to the manufacturer’s instructions.

### 4.3. Polymerase Chain Reaction, Sequencing, and Database Searching

The bacterial diversity was established by sequencing the PCR amplicons generated using the primers 27F (5'-AGAGTTTGATYMTGGCTCAG-3') and 518R (5'-ATTACCGCGGCTGCTGG-3'), which specifically target the highly variable V1–V3 region of the 16S gene [31]. Fungus-specific primers designed for 454 pyrosequencing were used to PCR-amplify an approximately 500-bp region of the 18S rRNA gene using the primers EF4a (5'-GGAAGGGRTGTATTTATTAG-3') and fung5a (5'-GTAAAAGTCCTGGTTCCCC-3') [32]. Amplicons were then column-purified using a QIAquick PCR Purification Kit (Qiagen, Valencia, CA, USA), quantified using a NanoDrop 2000 Spectrophotometer (Thermo Fisher, USA), and normalized for the future use in the emulsion-based clonal amplification and Ion-Torrent sequencing. Sequencing was performed on an Ion Torrent PGM™ (Life Technologies, Foster City, CA, USA) using an Ion PGM™ Template OT2 400 Kit (Life Technologies, Foster City, CA, USA) and an Ion 314™ chip (Life Technologies, Foster City, CA, USA) as per the manufacturer’s protocols.

### 4.4. OTU-Based Sequence Analysis

A module of the CD-HIT suite (http://cd-hit.org) was used for clustering reads. The workflow engine that manages the succession of steps and their dispatch to grid nodes was implemented in Python using YAP (http://github.com/shpakoo/YAP). An identity threshold of 97% was used to identify OTUs at approximately the species level [32].

### 4.5. Taxonomic Classification of Representative OTU Reads

The final set of representative reads was taxonomically classified using MOTHUR version (http://www.mothur.org/) of the Ribosomal Database Project (RDP) classifier and RDP training-dataset Number 6 that is normalized to contain six taxonomic levels for each sequence. A similar approach was used for classifying the 18S sequences, except that they were classified using an ARB program package and BLAST searches against the current NCBI nt database [32].

### 4.6. Anatomical Examination and Quantification of Vessel Occlusions in Stems

To determine the vessel-occlusion number, freehand transverse sections were cut from each sample, mounted in water on a coverslip, and examined by light microscopy (BX51, Olympus, Tokyo, Japan) [33,34,35]. For each section, five areas were randomly selected for the analysis.

### 4.7. Essential-Oil Extraction

The aforementioned wood samples were accurately weighed (10 g), dried, and powdered, after which the essential oil of the samples was extracted as previously described [19]. The distillates were dried over anhydrous sodium sulfate and stored at −20 °C until use in analyses.

### 4.8. Gas Chromatography-Mass Spectrometry Analysis

The composition of essential oils and sesquiterpenes obtained from *A. sinensis* was determined by GC-MS, which was performed using a Varian 450 gas chromatograph (Varian, Harbor City, CA, USA) equipped with a VF-5MS capillary column (60 m × 0.25 mm i.d., film thickness 0.25 μm) and a Varian 300 mass spectrometer equipped with an ion-trap detector in the EI mode at 70 eV in the m/e range of 10–550 amu. The GC-MS conditions and sesquiterpene compounds identification were performed according to our previous procedures [18].

### 4.9. Data Analysis

The average data obtained from five saplings (*n* = 5) was statistically analyzed. The significant differences among datasets were evaluated using Duncan’s multiple-range test.

## 5. Conclusions

The characterization of wounded trunks originated from *A. sinensis* induced by BCD and Agar-Wit method has very high similarity with that of agarwood induced by PTP, both in chemical composition and vessel-occlusion formation. However, no substantial variation was observed either in fungal and bacterial enrichment and diversity or in the relative abundances of taxa in trunks wounded by BCD and Agar-Wit. This suggests that the wounding without invasion of external microbes can induce agarwood formation. To our knowledge, this is the first report that wounding in the absence of variations in microbial communities can induce agarwood formation in* A. sinensis*.
